# Experimental Studies and Numerical Simulations of FRP Poles Failure in the Area of Inspection Hole

**DOI:** 10.3390/ma16062238

**Published:** 2023-03-10

**Authors:** Filip Broniewicz, Tadeusz Chyży, Krzysztof Robert Czech

**Affiliations:** Department of Geotechnics and Structural Mechanics, Bialystok University of Technology, 15-351 Bialystok, Poland; t.chyzy@pb.edu.pl (T.C.); k.czech@pb.edu.pl (K.R.C.)

**Keywords:** composite structures, GFRP lighting poles, flexural behavior, FEM analysis

## Abstract

Glass fiber-reinforced polymer (GFRP) utility poles are becoming more widespread in European countries. To ensure the integrity and safety of poles, it is necessary to carefully examine their structural features. The purpose of this paper is to present the numerical model of a column made with the engineering simulation software ANSYS and to compare the experimentally determined values of the stresses that lead to column failure close to the inspection hole with the results obtained using the numerical model. The critical buckling and failure loads for GFRP poles, as well as the associated modes of failure, were correctly predicted by the finite element method used in this study. Failure occurred in the middle of the inspection hole’s longer edge at a stress level of 220–250 MPa. A comparison of the stress using the ANSYS simulation software that led to the destruction of the column with those measured experimentally using strain gauges revealed a good agreement between their values.

## 1. Introduction

Composite poles are becoming more and more significant in the lighting sector. Concrete and metal poles still account for the vast majority of investments, but they are susceptible to the negative impact of environmental conditions. Greater durability and resilience to the weather conditions are qualities of composite materials. Investors have shown a great interest in composite poles for this reason, as well as the possible economic benefits.

Because GFRP composite columns are a novel and contemporary structural component, it is not yet entirely established how to determine their load capacity. The anisotropy of the material caused by the method of manufacturing the composite from polymer resin and glass fibers causes difficulties in determining the stresses. The freedom of their mutual arrangement and the different strengths of the components that make up the composite preclude the application of strength standards that are common to isotropic materials. In addition, there remains the problem of evaluating failure models, e.g., by buckling or loss of local stability, which depend on the composition of the composite material, as well as the shape and dimensions of the structure.

The search for an alternate material for building lightweight poles and masts was motivated by the desire to get rid of issues such as corrosion and to create a system that required no maintenance. A composite structure might significantly reduce total costs by forgoing the need for galvanizing and corrosion prevention over its lifespan. Investor interest in composite poles has grown significantly due to this, in addition to possible economic advantages.

The objective of this study was to employ the finite element method to develop new design guidelines that satisfy both the ultimate limit state and the serviceability limit state requirements, in accordance with European standards relevant to GFRP pole design, EN 40-7 [1] and EN 40-3-x [2,3,4].

The development of FRP (fiber-reinforced polymer) poles for use in electrical transmission and distribution networks has been the subject of several articles. The major goal of this research project is to examine the full-scale flexural behavior of tapered poles made of glass fiber-reinforced polymer (GFRP) in order to improve design and suggest improvements to the manufacturing process. When compared to traditional materials, the high specific strength and low stiffness of GFRP elements are their greatest distinguishing characteristics. Because of local or global buckling events, numerous elements fail as a result.

An analytical solution to the problem of local buckling of the chords and webs of composite beams and columns by taking into account the interaction between the chord and the web have been the subject of many studies [5,6]. According to the results of these studies, local buckling should be taken into account for thin-walled beams and columns. Finding the critical load of local wall buckling is essential for forecasting the column’s ultimate compressive and bending strength because local instability of the column wall causes member failure.

The behavior of scaled FRP models of transmission poles under cantilever loading conditions was studied by Zhi-Min Lin [7]. The four test subjects had round prismatic hollow cross-sections. The poles had a 6 mm thick wall and a 76 mm outer diameter. These were made by filament winding circularly arranged strips of pultruded sheet material. The test findings showed that the FRP poles behaved linearly up until failure.

Correia et al. [8] found that, as a result of the GFRP’s low Young’s modulus and high strength, excessive deformation and local and global buckling events, rather than material strength constraints, frequently determine the beam’s structural integrity. In addition, the low shear-to-Young’s modulus ratio implies that the shear deformation’s contribution is substantial, particularly in stocky elements.

Vito et al. [9] used the finite element method to analyze the strength and failure modes. One of the findings was that a GFRP shaft is between 37% and 80% lighter than a steel shaft, while still being safe and functional. For GFRP utility poles, a similar study [10] was carried out to determine the ideal cross-section dimensions and meet the ASTM strength standards.

In FRP profiles, local buckling was examined by Pecce and Cosenza [11]. Local buckling of the flanges was seen in several experimental results in compression and bending. Pecce and Cosenza presented their findings along with a summary of the critical stresses. The introduction and validation of a numerical model using the finite element method (FEM) involve contrasting the numerical outcomes with those from experiments. This finite element model was used in a broad parametric analysis to pinpoint a buckling curve for the flange’s local buckling.

The local buckling analysis of open and closed thin-walled section FRP composite beams and columns was also conducted by Kollar [12]. For bent box-, I-, C-, Z-, and L-members as well as for axially loaded members, explicit expressions have been devised. The width and the bending stiffnesses of each wall section were specified in these straightforward, plain formulas. The usefulness of the method was confirmed by the numerical examples.

The influence of openings in the cylindrical shells on their strength was studied by different researchers [13,14,15]. Investigation of the openings in steel wind turbine shaft show that presence of the opening leads to a strength reduction of 24%. Moreover, the critical load has the lowest value when the opening is on the compression side of the shell.

Ataş [10] used a three-dimensional progressive damage model (PDM) to predict the open hole compressive strength and damage mechanisms of various carbon fiber-reinforced plastic laminates. The impact of the Hashin stress failure criterion and maximum stress criterion on the PDM calculations was highlighted. Regarding the beginning of matrix damage, ultimate strength, and fiber damage pattern at the ultimate load, the maximum stress criterion showed greater overall correlation with the experimental data. The Hashin criteria predicted a scattered fiber damage pattern that included fiber tensile damage as a result of the shear stress contribution.

Metiche et al. [16,17] conducted a full-scale flexural testing on fiber-reinforced polymer (FRP) poles. Full-scale flexural testing was carried out using a novel test setup that was created and constructed in accordance with the recommendations of the ASTM-D4923-01 and ANSI-C136.20 standards. The study analyzed the varied geometry of the FRP columns, the type of fiber, the presence of the holes, and their location (compression side versus tension side). According to experimental findings, the use of low-linear-density glass fibers can increase the ultimate load bearing capacity by up to 38%. Additionally, the placement of the hole on the compression side as opposed to the tension side increases the ultimate load bearing capacity for 5.4 m FRP poles by up to 22% while having no discernible effect on 12 m poles. The layup sequence and the stress states created around the hole are primarily to blame for this.

A full-scale experimental investigation and structural assessment of GFRP poles were also carried out by Broniewicz et al. [18]. According to the study, the column deformed linearly until it was on the verge of failing (near-failure zone). All of the tested poles failed in the same manner, i.e., by buckling of the long free edges of the inspection holes. This was due to a reduction in the cross-section of the poles at the height of the inspection holes and the low stiffness modulus of the material. The EN 40-3-3 [4] standard, which specifies how to determine the strength of composite lighting columns, does not provide for this type of pole failure The values of the ultimate bending moment (UBM), calculated for the columns in accordance with the methodology presented in PN-EN 40-3-3 [4], were significantly higher (2–3 times) than the UBM values obtained during experimental tests in each analyzed case.

A second-order shell element and first-order shear deformation theory were used in Saboori and Khalili’s [19,20] study, concerning a linear static analysis of tapered FRP transmission poles with circular thin-walled cross-sections. In the calculations, the material was modeled as an orthotropic laminate. In analytical studies, the effects of fiber type/kind and orientation, volume fraction, number of layers, and their shape on the behavior of conical FRP transmission poles were examined. The results of the numerical calculations carried out using ANSYS engineering simulation software were compared with the analytical results to ensure accuracy of the study. A good agreement between analytical and numerical study results was achieved. The maximum discrepancy of the maximum stress with respect to the beam theory was 5%.

FEM studies of cylindrical elements modeled in FEM software as shell structures were also presented in [21,22]. The authors investigated the stress concentration factors (SCF) in steel tubular connections reinforced with FRP under in-plane bending load. The influence of FRP layer number on the SCF was studied. They found out that the stress concentration factors decreased with the growth of the FRP layer number, for both tubular T/Y-connections and tubular X-connections. The authors also derived parametric formulas to determine the SCFs in these types of connections.

Urgessa and Mohamadi [23] also numerically analyzed FRP poles using a finite element method algorithm. In their study, FEM parametric analyses were presented to ascertain the effects of the geometric properties, fiber orientation, number of layers, and lamina thickness on tapered FRP poles As a result of the tests, it was found that the maximum stresses in the FRP composite pole increased with the increase in fiber orientation angle to 45° (relative to the axial direction), and then decreased with a further increase in fiber orientation angle to 60°. It was also found that, with the increase in the number of layers in the poles, the maximum deflection and maximum stress decreased. However, as the number of layers increased, the rate of reduction decreased. In comparison to the “baseline” FRP pole with eight layers of 0.5 mm lamina thickness per layer, there was no noticeable difference in the maximum deflection and maximum stress between FRP poles of the same overall thickness with four or six layers.

An interesting comparison of Puck and Hashin damage theories in the case of modeling carbon fiber thermoplastic polymer was conducted by Ud Din et al. [24]. The phenomenon of adhesive wear in unidirectional laminate was studied. Different damage theories have been implemented in FEM software to predict the failure induced by adhesive wear and transverse compressive stresses. The 3D Puck theory was identified as more accurate, mostly due to its capability to account for the increase in shear strength with the increase in compressive stresses.

In [25], the authors examined the impact of the base plate dimensions and the location of the inspection hole on the load capacity of GFRP columns. In the first stage, tests were carried out for four sizes of the base plate (widths from 250 to 500 mm) and three different plate thicknesses (10 mm, 15 mm and 20 mm). The tests revealed no noticeable relationship between the base plate’s size and thickness on the capacity of the poles because all of the columns crashed down due to a loss of local stability and breaking of the column wall close to the opening. In the second stage, the effect of the location and reinforcement of the opening on the GFRP composite column’s load capacity was examined. Tests were conducted on three columns with inspection hole covers on the compression, tension, and lateral sides and two columns without inspection hole covers, with the exception that one of the inspection hole covers was not reinforced and the other was reinforced with a steel ring. The test results were at least puzzling, as they indicated that poles without the inspection hole cover could withstand twice as much load as poles with the cover. In addition, the columns with inspection hole covers with and without reinforcement had similar load capacities.

## 2. Material Properties

Material tests were conducted to determine the mechanical characteristics of the laminate, from which the column’s shaft was made. These included tensile static and flexural tests. This made it possible to determine five separate material properties: *f_y_*—characteristic tensile strength, *E*_1_, *E*_2_—characteristic value of the modulus of elasticity in the longitudinal and transverse directions, and ***ν***_12_ and ***ν***_21_, corresponding to the appropriate Poisson’s ratios.

The tensile tests were performed in accordance with ISO 527-4 [26]. Its results allowed determining the material’s properties under static tensile stress, including its modulus of elasticity (*E*), tensile strength (*σ_m_*), and tensile failure strain (*ε_m_*). Five samples were cut out from each of the experimentally tested columns. The samples were prepared with their axes parallel to the element of a column shaft’s cone and were prepared to fit the dimensions of type 2 specimens according to [26]. The outer coating layer was sanded down, in order not to influence the thickness measurement of the specimens.

**Figure 1:**
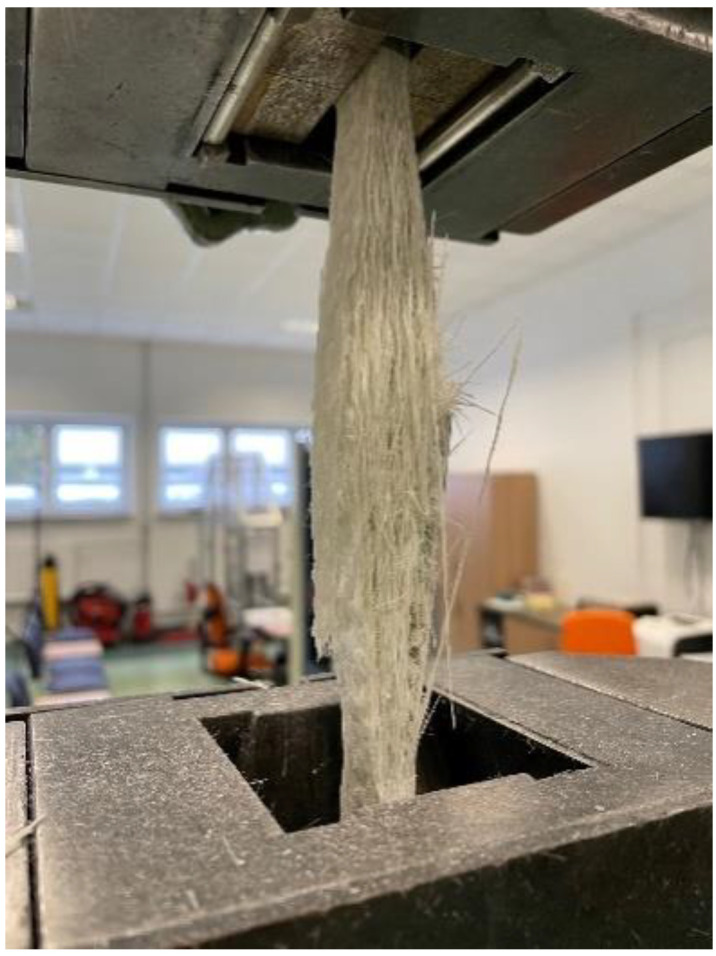
The specimen after failure—tensile test.

A BPS-HP004 machine manufactured by ZwickRoell in Ulm, Germany was used for the tensile tests. The tests were conducted with forces up to 100 kN (Figure 1). Table 1 shows the mean values and standard deviations for the static tensile test results. Utilizing linear regression of the experimental strength–strain curves corresponding to elongations between 0.05 and 0.25, it was possible to determine the estimated Young’s tensile modulus.

The specimens were loaded with a tensile force along the main axis at a constant speed of 2 mm/min until failure. A stress–strain curve defining the tested material was obtained by automatic recording of the tensile force and elongation of the measuring base of the tested sample.

All tested GFRP samples all failed in the same way, i.e., with fiber breakage, at maximum stress in the tensile test. Due to the surface detachment of the fibers and matrix, the failure manifested as an abrupt longitudinal delamination of the laminate in the center. As can be seen in Figure 1, the outcome was that the broken fibers formed a divergent and fan-like shape. The results for *E*_1_ and *E*_2_ were comparable, which allowed drawing the conclusion that the material was transversely isotropic. This type of material is standard in the production of composite elements that do not require an extensive design process and differentiation of material properties depending on the direction. Therefore, it was assumed in subsequent calculations that *E*_1_ = *E*_2_; only *E*_1_ is displayed in Table 1.

**Figure 2:**
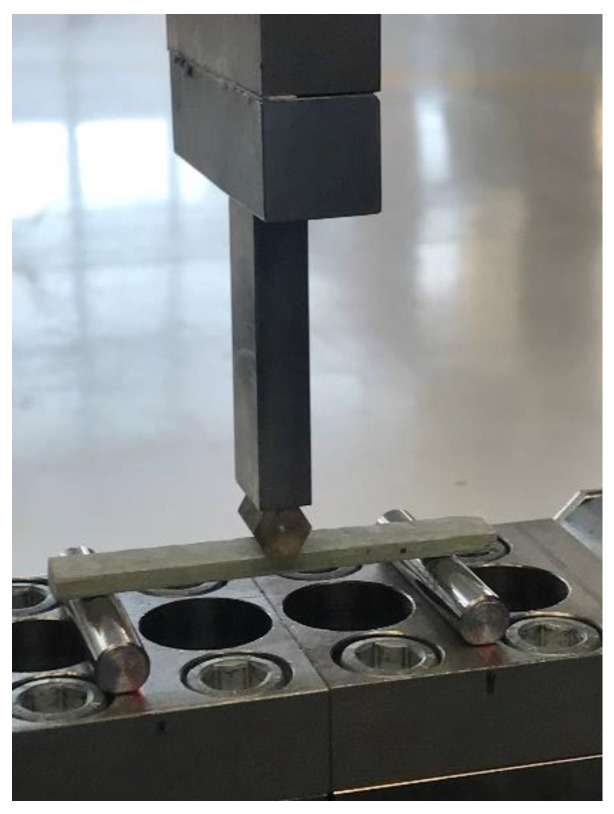
The specimen during bending flexural test.

A three-point bending flexural test (Figure 2) was used to evaluate the laminate’s bending characteristics. The tests were conducted in compliance with the EN ISO 14125 [27] standard’s requirements. Five samples were cut out from each of the experimentally tested columns. The samples were cut out with their axes parallel to the element of a column shaft’s cone and were processed in accordance with class II specimen dimensions according to [27]. The outer coating layer was sanded down, as in the tensile test.

The samples were supported with rolling supports. Then, the bending behavior of the GFRP material was examined by applying a bending force to standard specimens at a constant speed until fracture. The obtained results made it possible to calculate the bending strength *σ_fM_* and the bending stress at break *σ_fB_.* All of the specimens responded elastically under loading until they failed. The characteristic values of the flexural strength *σ_fM_* and flexural stress at break *σ_fB_* are presented in Table 2.

## 3. Cantilever Beam Static Bending Test

The purpose of the experimental study was to better understand the mechanism of column failure caused by the static load. This included an analysis of the column’s failure type, the location of the fracture, and the level of stresses present in the column at the moment of failure. Experimental testing was carried out using a dedicated test stand and multichannel measurement system for monitoring and recording strains of structures under load.

The lighting poles were produced with the use of the hand layup technique. The pre-impregnated layers were used. They were arranged as woven fabrics and chopped fiber sheets allowing for transverse isotropy of the laminate. There were five layers in total, including three woven ones. After the preparation of the laminate, a gel coating was applied to improve the appearance of the column.

The test stand was constructed in compliance with EN 40-3-2 [3] recommendations. It consisted of a 600 × 800 cm steel support block, a load-setting mechanism, and a sliding support with a set of bearings that allowed the end of the column to move freely. The use of the sliding support was intended to eliminate the additional load from the self-weight of the column.

**Figure 3:**
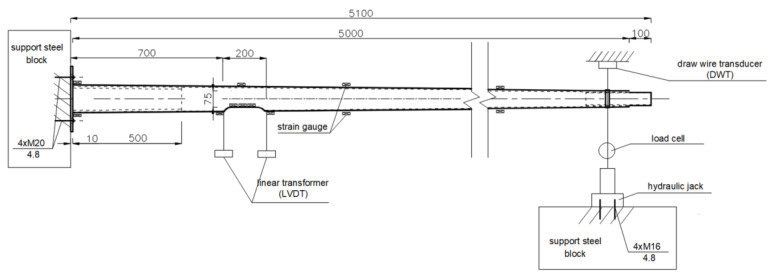
Full-scale test setup for 5 m poles.

The column was placed horizontally on a test stand and fixed to the support block with the use of four M20 anchor bolts. The load application system consisted of a hydraulic cylinder connected to the free end of the column. In accordance with the standard recommendations, the load was transferred in the horizontal plane, in the direction perpendicular to the column axis, at a distance of 0.5 m from the top of the column. The test stand is schematically shown in Figure 3.

An electronic measurement system by Hottinger Baldwin Messtechnik GmbH (Darmstadt, Germany) consisting of four interconnected 16 bit Spider8 devices (four- and six-channel) was used to measure the actual strains in selected places of the columns. All devices used in the measurements were equipped with carrier-frequency modules of the SR55 type, which enable electronic recording of various types of mechanical parameters with a sampling rate of up to 9600. The applied measurement system ensures simultaneous measurement of strains in 20 channels. The configuration of the measurements and the recording of the acquired signals were carried out using the Catman Easy software dedicated for Spider8 measuring devices (version 5.0.2, Hottinger Baldwin Messtechnik GmbH, Darmstadt, Germany), which was installed on an HP zBook 15 G3 mobile workstation. Strain measurements were carried out in a half-bridge arrangement and sampling rate of 10 Hz using 350 Ω TENMEX strain gauges with a measuring base of 5 mm (TF-5/350 type).

**Figure 4:**
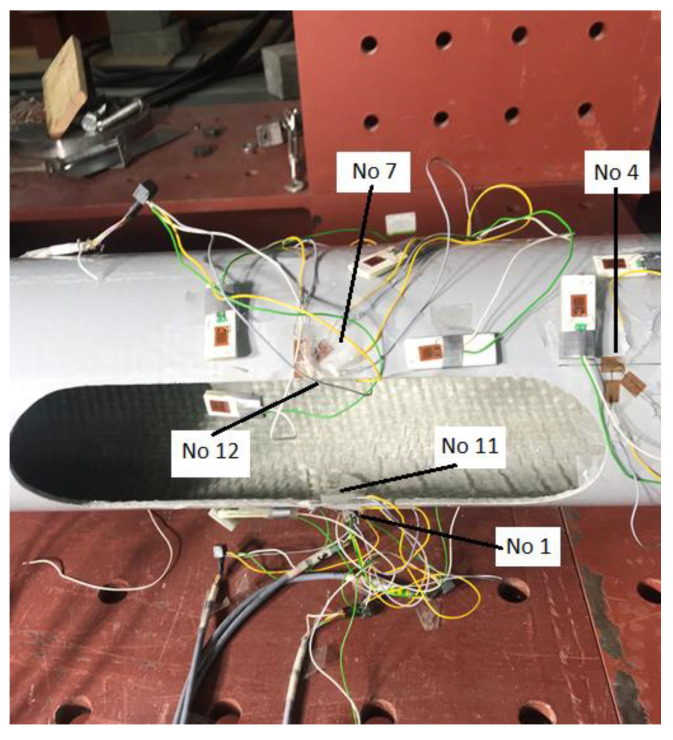
Locations of individual strain gauges.

Strain gauges have been placed near the inspection hole. Their task was to measure the strains occurring in the vicinity of the inspection hole during the loading of the column. The tested column was positioned so that the inspection hole was on the compression side. On each pole, six strain gauges were mounted around the inspection hole. Figure 4 illustrates how the strain gauges are positioned. To assess the problem of local buckling in this area, strain gauges were mounted in pairs on both sides of the laminate in the middle of the height of the inspection hole.

Figure 4 illustrates the locations of individual strain gauges:

- Strain gauge no. 1—in the middle of the length of the lower side of the inspection hole of the column (from the outside),
- Strain gauge no. 4—in the corner of the upper side of the pole inspection hole (from the outside),
- Strain gauge no. 7—in the middle of the length of the upper side of the inspection hole of the column (from the outside),
- Strain gauge no. 11—in the middle of the length of the lower side of the pole hole opening (from the inside),
- Strain gauge no. 12—in the middle of the length of the upper side of the inspection hole of the column (from the inside).

When testing composite poles, it is crucial to use the correct kind of gauge because problems, such as inaccurate strain measurements, can arise from a lack of heat dissipation from the strain gauge into the composite. Strain gauges with a higher resistance produce less heat for the generated voltage; hence, 350 gauges were considered to be the best option for composites. The choice of glue was carefully considered, and proper preparation of the sample prior to application of the adhesive reduced the likelihood of test failure. The gauge length of the strain gauges was much greater than the inhomogeneity of the material, which ensured that the measured strain is representative of the material sample.

**Figure 5:**
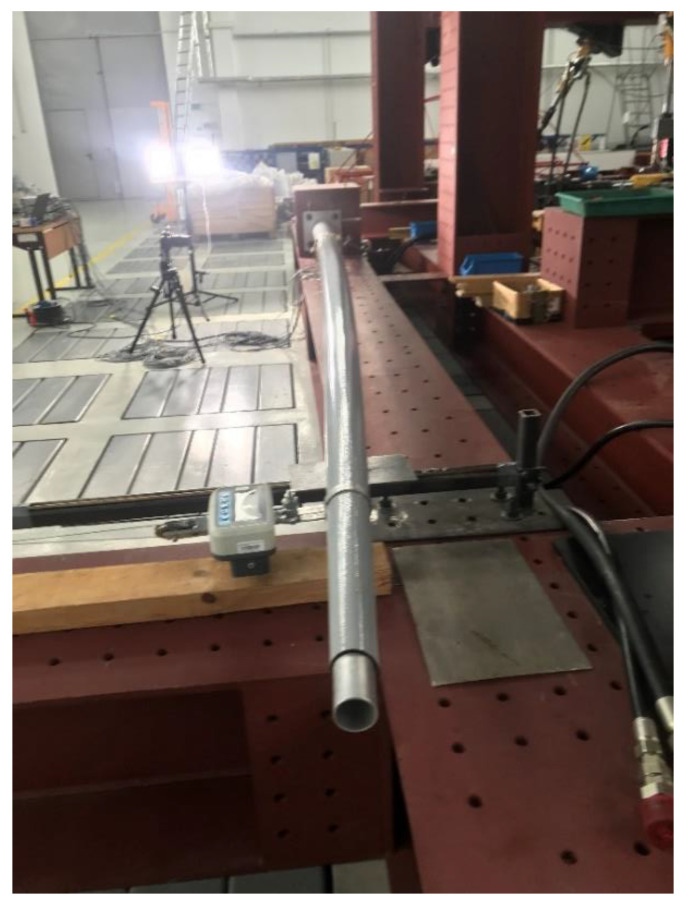
View of pole no. 5/2 mounted on the test stand.

The load was applied pointwise to the top of the column in a transverse direction relative to the test stand (Figure 5). The load on the column was gradually increased every 10 N until failure (measurements were preceded each time by a preload of approximately 100 N and a release cycle). During loading, strains and horizontal deflection of the column were measured. The moment of column failure was characterized by a sudden decrease in the load value and a large increase in horizontal deflection. In addition, macroscopic damage to the laminate layers could be observed.

**Figure 6:**
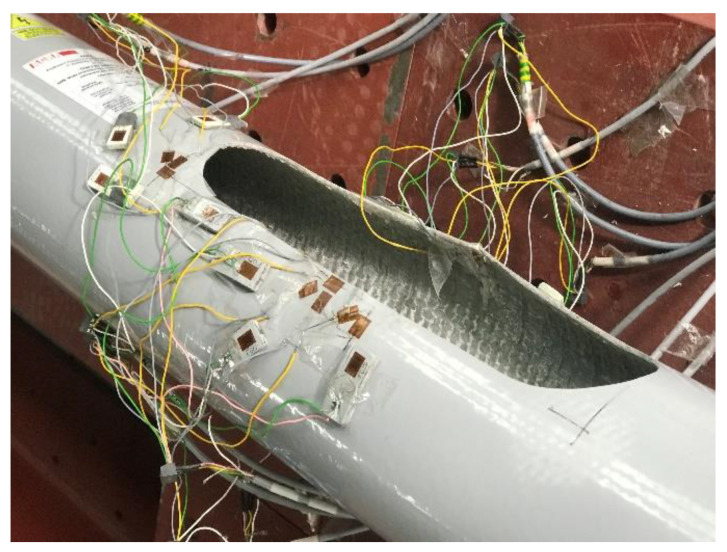
Visible damage to pole no. 5/2 in the area of the inspection opening.

In total, four poles were tested. There were two 5 m poles and two 9 m poles. The dimensions of tested poles and the experimental failure load values are summarized in Table 3. All columns were damaged by local buckling of the longer side of the inspection opening, which resulted in delamination and cracking visible in all layers of the laminate. A crack approximately 3 cm long could be observed symmetrically on both sides of the inspection hole (see Figure 6).

## 4. Finite Element Model

**Figure 7:**
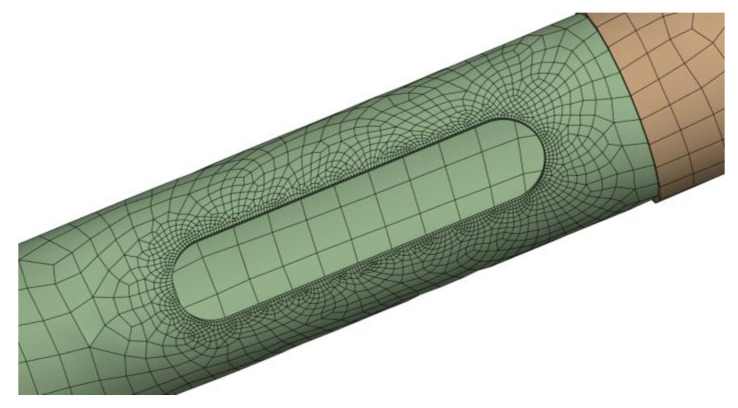
Mesh densification in the area of inspection hole area.

In order to construct geometrical models of columns to be used for numerical analyses using the engineering simulation software ANSYS, both data from material testing and measurements of column diameters were employed. The structure type for the FEM model was chosen to be a shell. The column shaft was designed in the shape of a truncated cone. An inspection hole with dimensions corresponding to the actual dimensions was also modeled. The increase in stiffness of the lower part of the column due to its fixing on the steel flange was reflected in the increased thickness of the poles in the lower 1.0 m section, starting from the base of the column. Four-node SHELL-181 finite elements, with six degrees of freedom per node (three degrees of freedom for translation $u_{X},u_{Y},u_{Z}$ and three degrees of freedom for rotation $\theta_{X},\theta_{Y},\theta_{Z}$), were used. Since nonlinear analysis requires correct estimation of stresses in the middle layer of the laminate, the KEYOPT(8) = 2 option was employed. The side length of the finite element was 4 cm. In the vicinity of the inspection hole, the finite element mesh was densified to 3 mm, allowing for a more precise mapping of the high-stress gradient there (Figure 7).

A total of 2450 finite elements (2340 nodes) were used for the numerical modeling of 5 m high columns. For 9 m columns, the number of finite elements increased to 5400 (5250 nodes). The continuity of the mesh between the model parts was respected with the use of shared topology.

The material chosen for the model was a laminate made of layers of glass fiber composite and epoxy resin (Table 4). The material was modeled at the mesoscale level; therefore, data about the properties of each lamina, its orientation, and the layup sequence were provided. The data needed to model the material forming the columns were obtained from material tests. The material exhibited isotropic features in the plane of the laminate. It was linearly elastic with a constant modulus of elasticity. This material was assigned to all elements forming the column.

Due to the layered nature of the material, the column was modeled as a laminate with the possibility of strain and stress analysis at the level of a single layer. The laminate was generated using the ACP module, a component of the ANSYS software. It allowed for the arrangement of layers that replicated the actual layup sequence in the laminate forming the pole.

Normal stresses parallel to the axis of the strain gauges were calculated using the strain gauge indications. The size of the finite element near the hole was smaller than the size of the strain gauge; therefore, the averaged stresses from the area of the size of the strain gauge, including several finite elements, were taken for comparison. The stress state in the vicinity of failure area was simple. The only significant stress value was normal stress parallel to the longer edge of the opening. The other stress values were much smaller and negligible. Therefore, the maximum stress criterion was adopted as the failure criterion, due to its simplicity. It states that the laminate layer fails when at least one of the stresses in the laminate reaches the strength obtained in material tests. The destruction of the element occurred when the following conditions were met:

(1) $$\sigma_{X}>F_{xT}\;for\;\sigma_{X}>0\;,x=\left\{1,2,\ldots ,6\right\},$$

(2) $$\sigma_{X}>F_{xC}\;for\;\sigma_{C}>0\;,x=\left\{1,2,\ldots ,6\right\},$$

where $\sigma_{X}$ is the stress value, $F_{xT}$ is the tensile strength of the composite in the *x*-direction, and $F_{xC}$ is the compressive strength of the composite in the *x*-direction.

## 5. Validation of FEM Model

**Figure 8:**
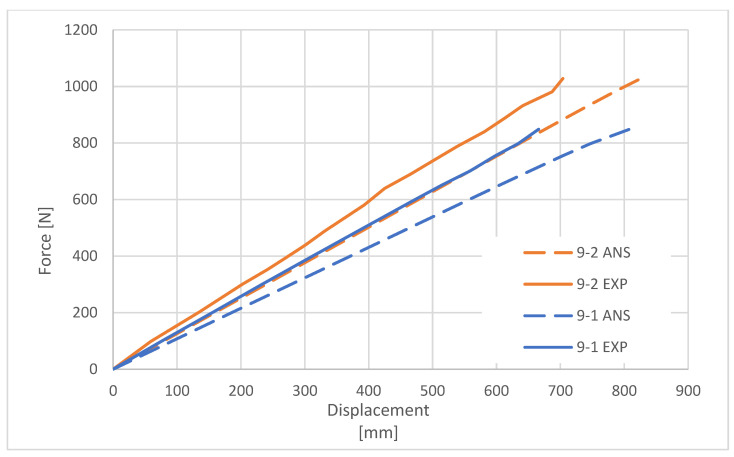
Load–deflection curves for 9 m long poles.

**Figure 9:**
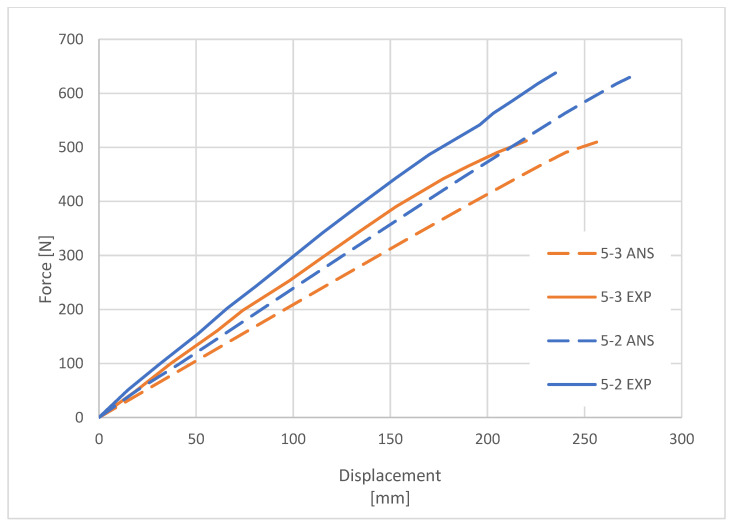
Load–deflection curves for 5 m long poles.

The purpose of the comparative analysis was to verify the accuracy of the FEM model. By comparing the deformation characteristics of the columns obtained during the experimental tests with the characteristics determined on the basis of the numerical simulations in ANSYS software, the correctness of the numerical model of the composite columns was confirmed (Figure 8 and Figure 9).

The curves presented above show a nonlinearity before the ultimate load was reached, which suggests a slight loss in flexural stiffness of the pole. This was most likely caused by the stresses that result from the applied moment and tend to flatten or ovalize the cross-section.

Parametric and nonparametric tests according to ISO 2602 [28] were run to examine the agreement of the curves. For the variables normally distributed within each group, a *t*-test was performed as a parametric test, and the variation in results in the two groups is not significantly different. The *Shapiro–Wilk (S–W)* test was used to determine whether the distribution was normal. The *F*-test, *Levene test, and Brown–Forsythe* test were used to confirm the equality of variances assumption. The remaining cases were subjected to nonparametric testing using the *Mann–Whitney U* test. There are no significant differences between the curves if the *p*-value from the *t*-test or the *Mann–Whitney U* test is greater than the specified significance level of α = 0.05. Therefore, the validation can be considered successful. The results of the analysis are shown in Table 5.

## 6. Measurement Data Analysis

**Figure 10:**
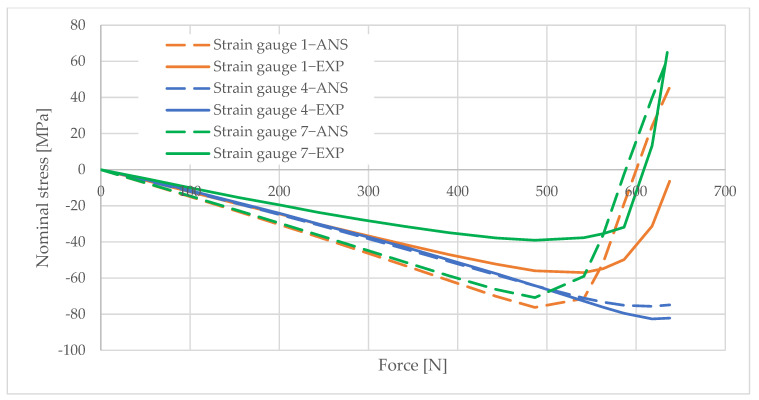
Pole 5/2—values of normal stress readings from strain gauges no. 1, 4, and 7.

**Figure 11:**
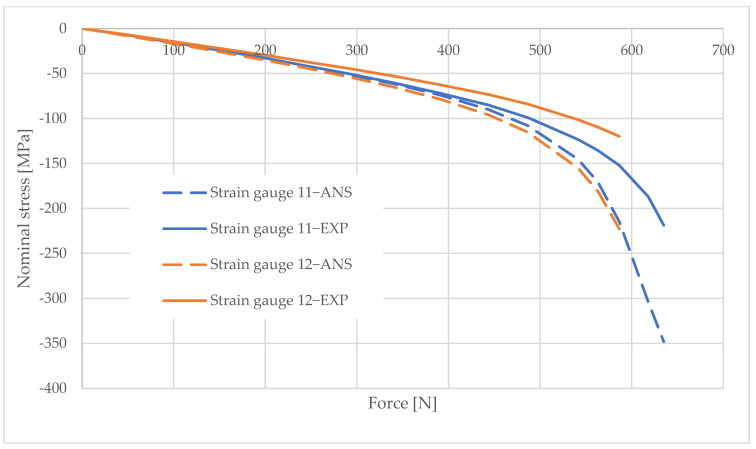
Pole 5/2—values of normal stress readings from strain gauges no. 11 and 12.

**Figure 12:**
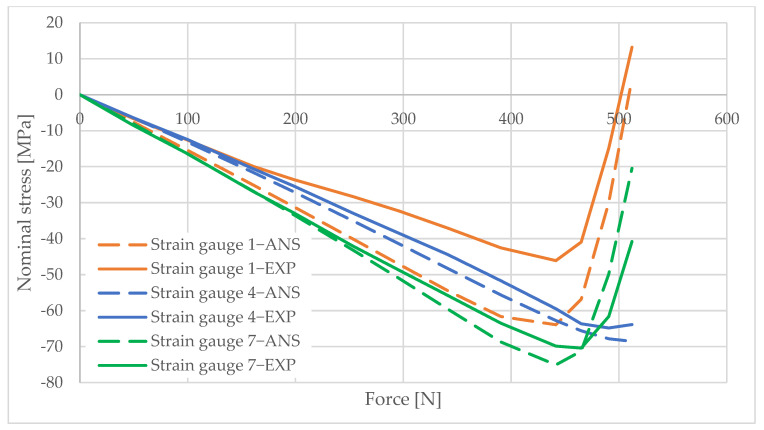
Pole 5/3—values of normal stress readings from strain gauges no. 1, 4, and 7.

**Figure 13:**
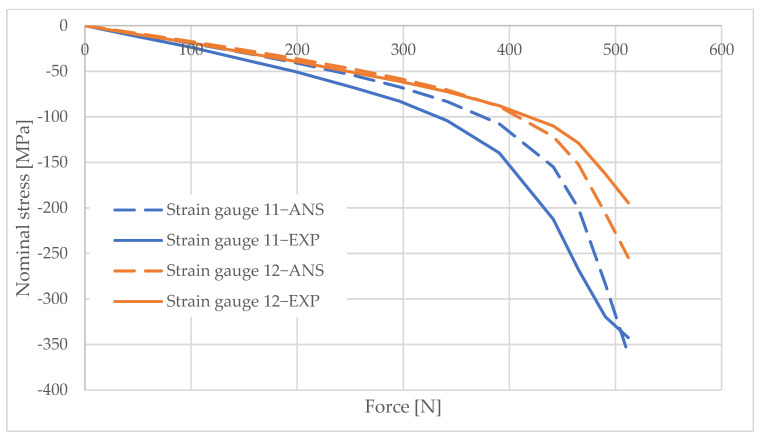
Pole 5/3—values of normal stress readings from strain gauges no. 11 and 12.

**Figure 14:**
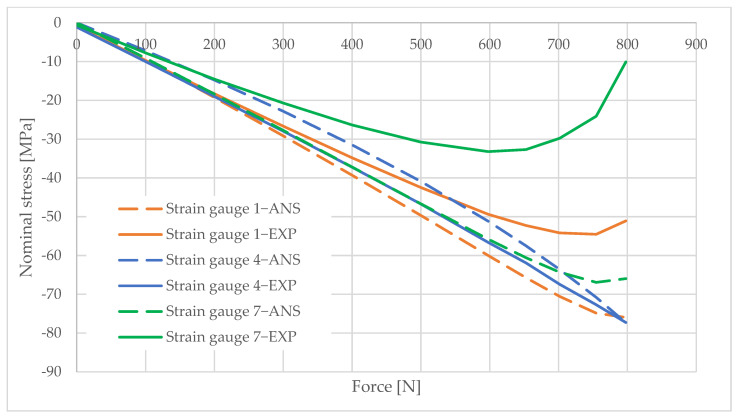
Pole 9/1—values of normal stress readings from strain gauges no. 1, 4, and 7.

**Figure 15:**
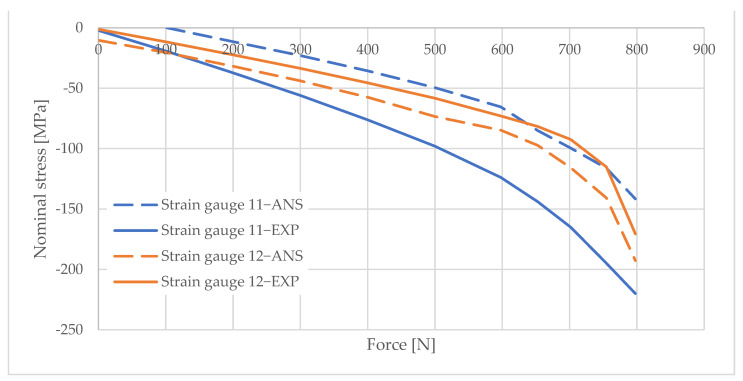
Pole 9/1—values of normal stress readings from strain gauges no. 11 and 12.

**Figure 16:**
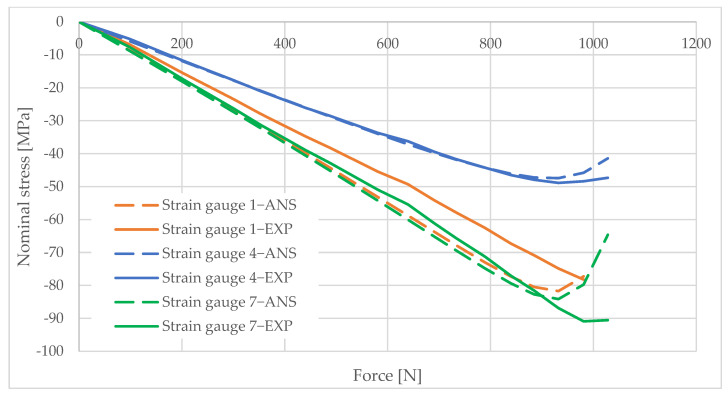
Pole 9/2—values of normal stress readings from strain gauges no. 1, 4, and 7.

Information obtained from strain gauge measurements shows the development of material deformation as the load increased. A validated numerical model was used to generate graphs illustrating the change in the stress values in the areas adjacent to the column opening. These graphs were created using the elastic modulus values obtained from the material tests and the applied load data. The graphs are shown in Figure 10, Figure 11, Figure 12, Figure 13, Figure 14, Figure 15 and Figure 16. The results of the statistical analysis are presented in Table 6 and Table 7.

For both parametric and nonparametric tests, the p-value was greater than the assumed significance level of α = 0.05. Thus, it can be concluded with 95% probability that the results obtained according to ANSYS were not significantly different from the experimental results.

By analyzing the curves, it can be shown that, in the pre-buckling phase, the stresses developing in the wall around the hole were linearly dependent on the applied external force. This was the elastic phase of the material’s behavior, and, after unloading the column, its deformation decreased to zero. In the pre-buckling loading phase, the strain values read from the strain gauges on the side of the opening were negative. This proves the presence of normal compressive stresses in the vicinity of the opening. At the time of buckling, the graph became nonlinear, which is typically due to the occurrence of second-order effects, causing an augmented increase in stress values. Second-order effects cause additional compressive or tensile stresses, depending on the laminate layer.

When comparing the stress graphs at the strain gauge locations glued on opposite sides of the laminate, a bifurcation of the function could be observed. This indicates the moment of loss of stability by the wall located near the door opening. The strain gauge located on the inside of the laminate (inside the column) showed an additional increase in compressive stresses. On the diagram for the outer side gauge, the influence of additional tensile stresses, reducing the stress values, can be seen. This was due to the wall of the long side of the column inspection hole buckling outward. The rapid increase in stresses in the inside lamina resulted in exceeding the critical compressive stress value and inner lamina failure.

## 7. Conclusions

This paper presented a study of the bending behavior of GFRP composite columns in the inspection hole area. Four columns were subjected to a static bending test. To determine the mechanical properties of the column material, samples were subjected to bending and tensile tests. Up to the breaking phase, the tests revealed linear plasticity of the column. The ANSYS engineering simulation software was used to perform the nonlinear numerical analysis of the conical GFRP composite columns. Layered composite shell elements were used to model the columns. The numerical analysis took into account the nonlinear behavior of the poles and included strength failure control through application of the maximum stress failure criterion. The results of the numerical analyses showed a high agreement with the results of experimental studies. In conclusion, it could be stated that the numerical model of the column reflected the behavior of the tested column under load and allowed determining the critical load related to the loss of stability of the column wall in the vicinity of the opening.

The main findings of the paper can be summarized as follows:

- For thin-walled GFRP poles, the critical buckling and material failure loads, as well as the corresponding failure modes, could be accurately predicted by the finite element method (FEM) used in this study,
- For modeling the behavior of FRP composite poles, the standard general-purpose shell element SHELL-181 in ANSYS was suitable and reasonably accurate,
- All the tested poles failed in the same way, i.e., by buckling of longer free edges of the inspection hole due to the low Young’s modulus of the column material and reduction in the column’s cross-section near the inspection hole,
- It was found that there was good agreement between the stress values calculated by the ANSYS engineering simulation software and the experimental strain values measured with strain gauges,
- Strain gauge readings showed negative strain values in the opening area, indicating the presence of normal compressive stresses in the pre-buckling loading phase. Subsequently, local buckling led directly to second-order effects and a rapid nonlinear increase in stresses,
- By using the developed FEM model, parametric tests could be carried out to predict the column buckling model in the vicinity of the opening.

## Figures and Tables

**Table 1:** Results of tensile tests (mean values and standard deviations).

Pole Height 5 m		
Specimen Number	Modulus of Elasticity *E,* MPa	Tensile Strength *σ_m_*, MPa
No. 1	20,870	225
No. 2	22,040	212
No. 3	21,870	245
No. 4	21,860	232
No. 5	19,720	266
Mean value	21,272	236
Standard deviation	983	20.5
95%	19,950	215
**Pole height 9 m**		
**Specimen number**	**Modulus of elasticity** ***E,* MPa**	**Tensile strength** ***σ_m_*, MPa**
No. 1	19,497	286
No. 2	19,069	304
No. 3	23,381	259
No. 4	19,607	281
No. 5	21,687	252
Mean value	20,389	283
Standard deviation	2008	18.5
95%	19,133	262.3

**Table 2:** Results of flexural tests (mean values and standard deviations).

Pole Height 5 m		
Specimen Number	Modulus of Elasticity *E,* MPa	Tensile Strength *σ_m_*, MPa
No. 1	16,520	268
No. 2	21,010	286
No. 3	17,660	274
No. 4	21,090	240
No. 5	22,520	267
Mean value	19,760	267
Standard deviation	2542	17
95%	16,748	245
**Pole height 9 m**		
**Specimen number**	**Modulus of elasticity** ***E,* MPa**	**Tensile strength** ***σ_m_*, MPa**
No. 1	17,950	314
No. 2	20,550	284
No. 3	18,290	304
No. 4	21,250	263
No. 5	16,260	260
Mean value	19,520	291
Standard deviation	1886	22.3
95%	18,001	267

**Table 3:** Dimensions of tested poles and the experimental failure loads.

Pole Identification	Length *L*, mm	Bottom/Top Diameters, mm	Mean Bottom Thickness, mm	Inspection Hole		
				Dimensions, mm	Location, mm	Positioning
5/2	5002	150/74	3.5	300 × 85	750	Compression
5/3	5001	150/74	3.25	300 × 85	750	Compression
9/1	9000	198/74	4.6	400 × 85	900	Compression
9/2	9000	198/74	5.0	400 × 85	900	Compression
	**Force at failure *F_B_*, N**			**Bending moment at failure *M_B_*, Nm**		
5/2	638			2391		
5/3	512			1912		
9/1	849			6456		
9/2	1028			7753		

**Table 4:** Configuration and number of fiber layers.

Part	Upper Part of the Pole	Lower Part of the Pole
Layup sequence	[90/0/90/0/90]	[90/0/90/0/90]*_T_*
Number of layers	5	10

**Table 5:** Testing differences between ANSYS model and experimental load–deflection graphs for tested poles.

Parametric Tests										
Pole No.	*S–W; p* ANSYS	*S–W; p* Experiment	*F-ratio*	*p*	*Levene’s*	*p*	*Brn–Fors*	*p*	*t*-test	*p*
5-2	0.946; 0.426	0.943; 0.381	1.358	0.560	0.575	0.454	0.572	0.455	0.861	**0.198**
5-3	0.953; 0.648	0.957; 0.706	1.380	0.585	0.501	0.486	0.469	0.499	−0.780	**0.221**
9-1	0.945; 0.563	0.934; 0.424	1.401	0.585	0.485	0.493	0.388	0.540	−0.701	**0.245**
9-2	0.966; 0.715	0.964; 0.672	1.329	0.564	0.517	0.477	0.486	0.490	0.855	**0.199**

**Table 6:** Comparison of normal stresses in the vicinity of the inspection opening from numerical simulations carried out in ANSYS software and experimental tests—5 m columns.

Pole 5/2—Parametric Tests										
Strain Gauge No.	*S–W; p* ANSYS	*S–W; p* Experiment	*F-Ratio*	*p*	*Levene’s*	*p*	*Brn–Fors*	*p*	*T*-Test	*p*
1	0.951; 0.497	0.920; 0.168	3.165	0.032	3.943	0.056	3.853	0.059	0.204	**0.420**
4	0.925; 0.210	0.905; 0.098	1.160	0.777	0.155	0.696	0.152	0.700	−0.109	**0.457**
12	0.913; 0.174	0.953; 0.612	3.070	0.053	3.491	0.073	2.221	0.148	−1.159	**0.129**
**Pole 5/2—Nonparametric tests**										
**Strain gauge No.**	***S–W; p*** **ANSYS**	***S–W*; *p*** **experiment**	***U***				***p***			
7	0.920; 0.170	0.770; 0.001	101.5				**0.372**			
11	0.854; 0.016	0.935; 0.260	116.5				**0.678**			
**Pole 5/3—Parametric tests**										
**Strain gauge No.**	***S–W; p*** **ANSYS**	***S–W; p*** **experiment**	***F-ratio***	***p***	***Levene’s***	***p***	***Brn–Fors***	***p***	***t*-test**	***p***
1	0.940; 0.460	0.961; 0.775	1.713	0.364	1.375	0.252	1.266	0.271	−1.291	**0.105**
4	0.923; 0.273	0.923; 0.276	1.107	0.863	0.067	0.798	0.060	0.808	0.243	**0.450**
7	0.953; 0.644	0.942; 0.487	1.137	0.828	0.201	0.658	0.159	0.693	0.109	**0.462**
12	0.886; 0.087	0.939; 0.448	1.731	0.355	0.829	0.372	0.362	0.553	0.342	**0.368**
**Pole 5/3—Nonparametric tests**										
**Strain gauge No.**	***S–W; p*** **ANSYS**	***S–W*; *p*** **experiment**	***U***				***p***			
11	0.854; 0.032	0.875; 0.602	77.0				**0.720**			

**Table 7:** Comparison of normal stresses in the vicinity of the inspection opening from numerical simulations carried out in ANSYS software and experimental tests—9 m columns.

Pole 9/1—Parametric Tests										
Strain Gauge No.	*S–W; p* ANSYS	*S–W; p* Experiment	*F-ratio*	*p*	*Levene’s*	*p*	*Brn–Fors*	*p*	*t*-test	*p*
1	0.918; 0.305	0.881; 0.108	2.013	0.286	2.353	0.141	1.677	0.210	−0.909	**0.187**
4	0.954; 0.694	0.942; 0.540	1.022	0.980	0.0002	0.992	0.003	0.960	−0.526	**0.374**
7	0.901; 0.188	0.877; 0.100	2.044	0.275	2.415	0.136	1.490	0.236	−1.111	**0.140**
11	0.961; 0.781	0.964; 0.814	1.640	0.448	1.015	0.325	0.943	0.343	1.088	**0.145**
12	0.952; 0.667	0.950; 0.647	1.227	0.753	0.114	0.739	0.103	0.749	0.661	**0.258**
**Pole 9/2—Parametric tests**										
**Strain gauge No.**	***S–W; p*** **ANSYS**	***S–W; p*** **experiment**	***F-ratio***	***p***	***Levene’s***	***p***	***Brn–Fors***	***p***	***t*-test**	***p***
1	0.934; 0.255	0.964; 0.706	1.200	0.720	0.289	0.595	0.234	0.632	−0.669	**0.254**
4	0.914; 0.115	0.926; 0.190	1.051	0.922	0.009	0.927	0.008	0.929	−0.028	**0.489**
7	0.935; 0.269	0.968; 0.788	1.053	0.909	0.0003	0.986	0.002	0.965	−0.114	**0.455**

## Data Availability

Data available on request due to privacy.
